# High variability in a mating type linked region in the dry rot fungus *Serpula lacrymans *caused by frequency-dependent selection?

**DOI:** 10.1186/1471-2156-11-64

**Published:** 2010-07-12

**Authors:** Ingeborg Bjorvand Engh, Inger Skrede, Glenn-Peter Sætre, Håvard Kauserud

**Affiliations:** 1 Microbial Evolution Research Group (MERG), Department of Biology, University of Oslo, P.O. Box 1066 Blindern, N-0316 Oslo, Norway; 2 Centre for Ecological and Evolutionary Synthesis (CEES), Department of Biology, University of Oslo, P.O. Box 1066 Blindern, N-0316 Oslo, Norway

## Abstract

**Background:**

The mating type loci that govern the mating process in fungi are thought to be influenced by negative frequency-dependent selection due to rare allele advantage. In this study we used a mating type linked DNA marker as a proxy to indirectly study the allelic richness and geographic distribution of mating types of one mating type locus (MAT A) in worldwide populations of the dry rot fungus *Serpula lacrymans*. This fungus, which causes serious destruction to wooden constructions in temperate regions worldwide, has recently expanded its geographic range with a concomitant genetic bottleneck.

**Results:**

High allelic richness and molecular variation was detected in the mating type linked marker as compared to other presumably neutral markers. Comparable amounts of genetic variation appeared in the mating type linked marker in populations from nature and buildings, which contrast the pattern observed with neutral genetic markers where natural populations were far more variable. Some geographic structuring of the allelic variation in the mating type linked marker appeared, but far less than that observed with neutral markers. In founder populations of *S. lacrymans*, alleles co-occurring in heterokaryotic individuals were more divergent than expected by chance, which agrees with the expectation for populations where few mating alleles exists. The analyzed DNA marker displays trans-species polymorphism wherein some alleles from the closely related species *S. himantoides *are more similar to those of *S. lacrymans *than other alleles from *S. himantoides*.

**Conclusions:**

Our results support the idea that strong negative frequency-dependent selection maintains high levels of genetic variation in MAT-linked genomic regions, even in recently bottlenecked populations of *S. lacrymans*.

## Background

A high allelic richness is maintained in some genetic loci due to negative frequency-dependent selection caused by rare allele advantage, which counteracts the effect of genetic drift (reviewed by Richman 2000). The MHC (Major Histocompatibility Complex) system of animals and the SI (self incompatibility) system in plants are well-known examples [1]. The mating type (MAT) loci of fungi, which governs the mating process, are also thought to be influenced by negative frequency-dependent selection because haploid mycelia possessing rare MAT alleles have higher chances for mating compared to those possessing frequent alleles [2,3].

Different types of mating systems occur in the fungal kingdom. Most basidiomycetes have a tetrapolar mating system where two separate gene complexes, MAT A and MAT B, govern the mating process. MAT A encodes homeodomain transcription factors, MAT-B encodes pheromones and pheromone receptors, and together they control mate recognition, clamp connection formation and pairing of nuclei in the formation of dikaryotic mycelium [4]. For mating to occur in tetrapolar species, different allelic versions must be present at both mating type loci. In homobasidiomycetes (i.e. basidiomycetes with a non-divided basidium), multiple alleles exist in the mating type loci and negative frequency-dependent selection can promote maintenance of a high richness of MAT alleles in populations of these fungi [5,6]. This rare allele advantage may also lead to 'trans-specific polymorphisms' because of extended coalescence times between alleles [7]. By classical mating studies it has been shown that a large number of MAT alleles are present in some taxa. In *Schizophyllum commune*, it has been estimated that around 160 A mating types exist in nature [5], while in *Coprinopsis cinerea*, the number of A mating type alleles was estimated to be 100 [8]. Although this high allelic diversity suggests that MAT would be an excellent target for the development of population genetic markers, MAT alleles have so far not been used in population genetics studies of Agaricomycetes (Basidiomycota). This is likely due to the fact that MAT alleles themselves are highly divergent in sequence, making it difficult to design universal primers.

It has been demonstrated that a conserved gene order (shared synteny) exists between the mating type genes and neighbouring genes in most Agaricomycetes [9]. One such locus is the gene encoding mitochondrial intermediate peptidase (*mip*), located close to the MAT A locus (less than 1 kb for *S*. *commune*, *Coprinopsis cinerea *and *C. scobicola*) in Agaricomycetes investigated [10,11]. Because of physical linkage, we may expect that this gene is indirectly affected by negative frequency-dependent selection acting on MAT, and therefore that high allelic richness will be maintained also in the *mip *region due to linkage disequilibrium. Accordingly, instead of targeting the mating types directly, which poses technical difficulties due to massive molecular divergence, targeting neighbouring 'non-mating type MAT-linked genes', such as *mip*, may yield proxies for analysing the allelic richness of mating types [9] and a source for highly variable markers.

In this study we analyse a genetic marker covering a portion of the *mip *gene, a neighbouring spacer region and the 3'-prime end of the mating type gene, homeodomain 1 (HD1), as a proxy to analyse the richness of mating types (MAT A) within the dry rot fungus *Serpula lacrymans *(Boletales, Basidiomycota). *Serpula lacrymans *is the most damaging destroyer of wood constructions in temperate regions. By using various presumably neutral genetic markers, we have previously shown that the dry rot fungus is divided into two main lineages that probably represent different species; one non-aggressive residing naturally in North America and Asia (var. *shastensis*), and another aggressive lineage appearing on all continents (var. *lacrymans*) [12]. Genetic analyses pinpoint mainland Asia as the origin of the aggressive form var. *lacrymans*, from where it has migrated worldwide to Europe, North- and South America and Oceania followed by local population expansions [12]. This recently spread lineage of var. *lacrymans*, probably spread by man in historic time, is known as 'the Cosmopolitan group' [12]. Little genetic variation occurs in the bottlenecked founder populations of var. *lacrymans *worldwide, while more genetic variation is found in the source population in mainland Asia, as well as in var. *shastensis *[12,13]. In accordance with this inferred massive recent expansion from a much smaller founder population, only a few (6) vegetative compatibility types (VC-types) have been detected in the European genetically depleted population of var. *lacrymans *[14]. Vegetative compatibility is a self-nonself recognition system used to separate own mycelium from other mycelia. Normally, one VC-type corresponds to one genet, but in genetically depleted populations, like in *S. lacrymans*, different genets can belong to the same VC-type. Higher genetic variation and a correspondingly higher number of VC-types have been detected in the Japanese indoor population, which probably was founded during an independent founder event from the Asian source population [13]. The morphospecies *Serpula himantioides*, which is used as outgroup in our analyses, is the sister taxon to *S. lacrymans *and includes several sub-groups that probably represent independent ('cryptic') species [12].

The aims of the present study are to use a non-mating type MAT-linked marker as a proxy to indirectly study the allelic richness of mating type A in a worldwide sample of the genetically deprived *S. lacrymans*, and to investigate whether the level of molecular variation at the MAT-linked loci is consistent with negative frequency-dependent selection. Furthermore, we analyse the allelic richness in a geographic context and investigate whether more variation occurs in natural, outdoor populations of var. *shastensis *and var. *lacrymans *compared to the founder populations of var. *lacrymans *that strictly appears in buildings. Finally, we evaluate whether the mating type linked marker can be used to separate closely related isolates of var. *lacrymans*.

## Methods

### Material

A total of 83 cultures and dried specimens of *S. lacrymans *and *S. himantioides *were included in this study (see additional file 1: Information about the analyzed material). DNA was extracted following a 2% CTAB (cetyl trimethylammonium bromide) miniprep method described by [15] with minor modifications: DNA was resuspended in 100 μL distilled sterile H_2_O at the final step of extraction.

### PCR

We first tested the primers MIP1F and MIP1R [16] on different *Serpula *strains. This primer set has successfully been used to amplify a part of the non-mating type MAT-linked *mip *gene in various other fungi [16]. Positive amplicons were obtained from a few isolates only (probably due to primer mismatch). Based on an alignment including partial *mip *sequences from three Boletales species [16], as well as three *Serpula *sequences obtained using the MIP1 primer set, we designed the new primers mip60F (GGMAAYCAYCACGAAGAYCC) and mip190F (TTCAGCCATCTATTYGGGTACGG). In order to maximize sequence length we employed an uneven PCR approach as described in [17], combining the primers mip60F and mip190F and twelve different RAPD primers [17]. Positive amplicons from different primer combinations were sequenced, and finally the new primers mip55R (GCGGACAAACAAGCAAAGTT) and mip82R (CTGAAGATGCTGGAGGAAGC) were designed based on the resulting alignments and further combined with mip60F and mip190F to amplify the partial *mip *region from all included isolates and specimens (Table 1). PCR amplification with primers mip60F or mip190F in combination with primers mip55R or mip82R was performed with the proofreading enzyme Dynazyme EXT DNA Polymerase (Finnzymes) with reactions containing 16.5 μl of 100× diluted template DNA, 1.5 μl each of forward and reverse primers (5 μM stocks), 2.5 μl of dNTPs (2 μM stock), 2.5 μl of Dynazyme EXT 10× reaction buffer, 0.5 μl Dynazyme EXT DNA Polymerase (25 μl total reaction volume). PCR reactions with Dynazyme EXT were performed with the following protocol: 2 min at 94°C; followed by 35 cycles of 30 s at 94°C, 45 s at 54°C, and 1 min at 72°C; followed by a 7 min extension at 72°C and an indefinite hold at 4°C. Based on the two genome sequences of *Serpula lacrymans *S7.3 and S7.9 (U.S. Government Department of Energy - Joint Genome Insitute) a new primer (Mip_ins3R; ACTCCGCTGAAGTCCACCTGC) was designed in an insert occurring in some allelic versions of the mating type linked marker. Mip_ins3R was combined with mip190F to reveal the presence of this insert (see below) using the same PCR conditions as above. We sequenced nine amplicons directly to assess the homology of the insert.

**Table 1:** Information about molecular variation in the four sequenced loci of *Serpula lacrymans*.

Locus	Group	Ecol^1^	#	S	k	π	Theta W	Tajima's D	Fu and Li's D*	Fu and Li's F*
MAT linked marker	*S. lacrymans*	N+B	116	182	19.4	0.048	34.17	-1.74	-3.45*	-3.22*
	var. *lacrymans*	N+B	95	183	23.1	0.049	35.70	-1.48	-3.42*	-3.10*
	var. *shastensis*	N	21	170	34.5	0.051	47.25	-1.37	-1.77	-1.93
	var. *lacr*. Asia	N	16	158	45.09	0.062	47.62	0.43	0.64	0.67
	var. *lacr*. Japan	B	27	132	25.76	0.049	34.25	-1.22	-1.28	-1.49
	var. *lacr*. Cosmo.	B	51	213	38.91	0.057	47.34	-0.9	-2.61*	-2.34
										
ITS	*S. lacrymans*	N+B	150	11	2.2	0.004	1.97	0.32	0.32	0.67
	var. *lacrymans*	N+B	126	3	0.6	0.001	0.56	0.18	-0.64	-0.44
	var. *shastensis*	N	24	2	0.3	0.0006	0.54	-0.89	0.84	0.42
	var. *lacr*. Asia	N	20	1	0.51	0.0009	0.28	1.43	0.65	0.98
	var. *lacr*. Japan	B	32	1	0.42	0.0007	0.25	1.04	0.59	0.82
	var. *lacr*. Cosmo.	B	74	2	0.05	0.0001	0.41	-1.42	- 2.71*	-2.71*
										
*gpd*	*S. lacrymans*	N+B	122	107	5.4	0.007	19.90	-2.36**	2.36*	0.38
	var. *lacrymans*	N+B	98	7	1.5	0.002	1.36	0.21	-0.55	-0.55
	var. *shastensis*	N	24	106	18.1	0.022	28.39	-1.45	1.84*	0.94
	var. *lacr*. Asia	N	8	5	1.61	0.0020	1.93	-0.76	-0.49	-0.61
	var. *lacr*. Japan	B	32	6	1.38	0.0017	1.49	-0.21	-1.15	-1.01
	var. *lacr*. Cosmo.	B	58	0	-	-	-	-	-	-
										
*tub*	*S. lacrymans*	N+B	146	30	7.07	0.018	5.40	0.90	1.25	1.34
	var. *lacrymans*	N+B	122	1	0.14	0.0004	0.19	-0.29	0.48	0.29
	var. *shastensis*	N	24	6	1.30	0.003	1.61	-0.57	-0.24	-0.39
	var. *lacr*. Asia	N	18	1	0.21	0.0005	0.29	-0.53	0.67	0.40
	var. *lacr*. Japan	B	32	1	0.35	0.0008	0.25	0.64	0.59	0.69
	var. *lacr*. Cosmo.	B	72	0	-	-	-	-	-	-

The information about ecology, molecular variation and deviations from neutral evolution in the four analyzed sequence loci are given for different sub-groups of *Serpula lacrymans*.

^1^N = nature, B = building, # indicates number of investigated sequences, S = number of segregating sites, k = average number of nucleotide differences, π = nucleotide diversity.

### Cloning and sequencing

We used a cloning procedure to separate different *mip *alleles co-amplified from the heterokaryotic isolates and specimens. Fragments were cloned with the TOPO TA Cloning kit (Invitrogen) using blue/white screening according to the manufacturer's manual. Positive colonies were subjected to direct PCR with the M13R/T7 primers with the same PCR conditions as described above. The resulting amplicons were sequenced using an ABI 3730 DNA analyser (Applied Biosystems, Foster City). We aligned the cloned sequences from each isolate/specimen manually in separate alignments using BioEdit 7 [18] (from three to twenty, see additional file 1: Information about the analyzed material), and two divergent alleles (when present) were separated. It is well known that artificial mutations and chimeric sequences can be obtained when using a clone-based approach. When more than two copies of each allele were present in the alignment, we considered polymorphisms occurring only within one single sequence (being 'autapomorphic') as mutations generated *in vitro *during PCR and discarded these. When only two copies of each allele were present we assumed heterozygosity at the sites in which the two copies differed. When only one allelic copy was present this version was accepted as is. Sequences from the internal transcribed spacer (ITS) nrDNA region and parts of the beta tubulin (*tub*) and glyceraldehyde-3-phosphate dehydrogenase (*gpd*) genes have previously been obtained from the isolates/specimens as specified in [12]. These sequences were used as comparisons with the *mip*-sequences analysed here. All sequences have been deposited in GenBank under the accession numbers given in additional file 1: Information about the analyzed material.

### Phylogenetic and statistical analyses

We established sequence alignments for the mating type linked marker *mip*, as well as ITS, *gpd *and *tub *and ran phylogenetic analyses on the two first datasets. 'Best-fit' evolutionary models were estimated for all analyses using the Akaike information criterion (AIC) as implemented in MrModeltest 2.3 [19]. The SYM+I+G model was specified as prior for the MAT linked data set while the HKY+I model for the ITS data set. Posterior probabilities were determined by MrBayes [20,21] twice by running one cold and four heated chains for 10 × 10^6 ^generations in parallel mode, saving trees every 1000th generation. A 50% majority rule consensus tree was used to calculate posterior probabilities including the proportion of trees gathered after the convergence of likelihood scores was reached. We run parsimony analyses using TNT [22], where Jackknife support values [23] were obtained using 1000 replicates. Jackknife support values above 50 were superimposed on the consensus tree from MrBayes.

Due to the dikaryotic stage of the isolates/specimens, heterozygous sites (with two nucleotides in same position) appeared in many of the *gpd*, ITS and *tub *sequences. In order to use the information in the heterozygous sequence sites and calculate more accurate estimates of molecular variation for these regions, haplotype datasets were constructed for the different DNA regions. For example, in a DNA sequence ('genotype') containing an 'Y' (=C/T) the two resulting sequence haplotypes will include either a 'C' or a 'T'. In sequences with more than one heterozygous site, the heterozygous phase was inferred using a procedure yielding the minimum number of alleles. In short, sequences homozygous at all sites or heterozygous at only one site (known putative haplotypes) are used as templates for inferring phase of sequences with multiple heterozygous sites. Hence, sequences with two or more heterozygous sites are, whenever possible, assigned to known putative haplotypes found in the sample of sequences. Alternatively, haplotypes requiring a minimum number of mutational steps are inferred. The procedure may underestimate the number of haplotypes if recombinant genotypes occur. Notably, descriptive statistics of level of polymorphism (such as π and k), as well as tests of neutrality (Tajima's D and Fu & Li's F and D tests) will not be affected since these statistics are based on polymorphic sites per se and not haplotypes. In ITS no more than one heterozygous site appeared per sequence. Nucleotide diversity π (average number of nucleotide differences per site), *k *(average number of nucleotide differences per sequence per site) and estimates of the population mutation parameter theta *θ *were calculated for each of the four sequenced loci using the program DnaSP version 4.50.2 [24]. To test for deviation from neutral evolution, we performed Fu and Li's D, Fu and Li's F and Tajima's D tests using DnaSP. The HKA test [25] was performed with the program HKA for all four loci (published by Jody Hey; http://lifesci.rutgers.edu/~heylab/ProgramsandData/Programs/HKA/HKA_Documentation.htm) with 1000 simulations.

To test whether the two alleles co-amplified from a single individual were more divergent than expected by chance, we calculated pairwise similarities of all alleles using Bioedit. This matrix was then used to calculate the mean similarity of alleles occurring within individuals, which was compared to the mean similarity of alleles across individuals, by a two-sample unequal variance t test using the software R [26]. This test was done independently for different groups of isolates; all isolates of *S*. *lacrymans *var. *lacrymans*, the cosmopolitan (Europe + North America + Oceania) group of var. *lacrymans*, European isolates of var. *lacrymans*, Japanese isolates of var. *lacrymans*, and all isolates of *S*. *lacrymans *var. *shastensis*.

## Results and discussion

### The MAT-linked marker

The sequences we obtained from the MAT-linked marker ranged from 718 to 810 bp. In the sequence alignment (860 bp), the first 285 positions constituted the 3' end of the *mip *gene, positions 286-832 a spacer region, and 833-860 the 3' end of the HD1 gene of MAT A. A total of 126 sequences of the MAT-linked marker were obtained from the 83 analysed dikaryotic isolates/specimens. In a dikaryotic basidiomycete it is expected that two different MAT A alleles occur in each isolate/specimen, but we obtained two alleles from only 53% of the analysed dikaryons. This could be due to (i) failure to detect both alleles due to limited number of cloned fragments (6-20) from each isolate, (ii) primer mismatch (or other reasons for PCR amplification failure), or (iii) that different MAT alleles share the same mating type linked marker (e.g. due to recombination and/or that the linked marker is more conserved than the adjacent MAT alleles). Notably, a comparison of the recently analysed genome sequences of *Serpula lacrymans *var. *lacrymans *(i.e. the two sequenced homokaryons S7.3 and S7.9, obtained from the same spore family), revealed that a large insertion (~18 kbp) occurred between *mip *and the mating type locus in one of the genomes (S7.9). Thus, a plausible explanation for why only one allele was amplified in a large proportion of the isolates is that this insertion occurs in many of the analysed isolates. Indeed, PCR amplification with one primer located within and one outside the insert gave positive amplicons from isolates where only one allele originally was obtained. Surprisingly, from two of the isolates we obtained three and four different alleles (see below).

### Molecular and allelic variation

**Figure 1:**
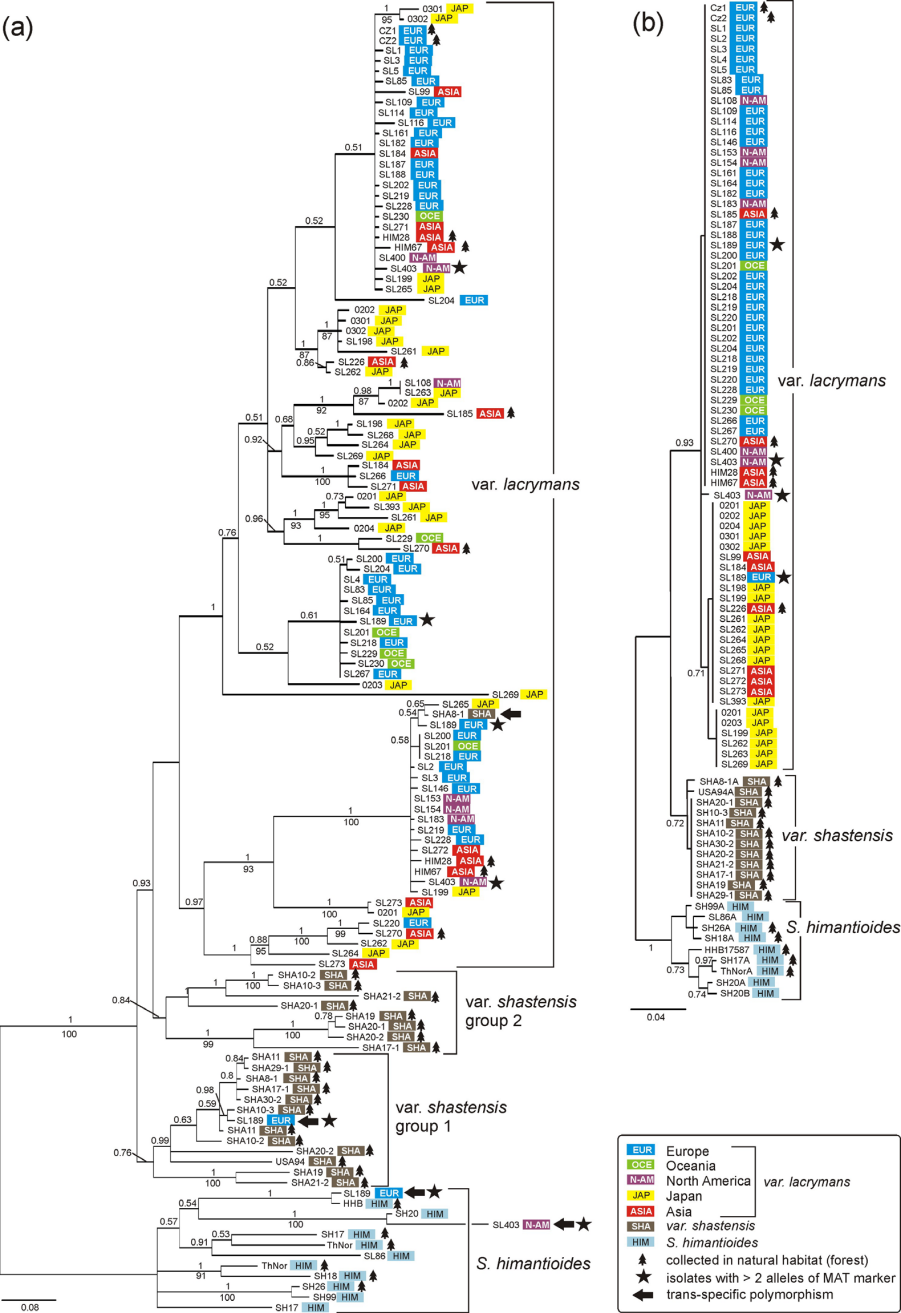
**Phylogenies of the MAT A linked marker and ITS**. 50% majority rule consensus trees obtained from Bayesian analyses of (a) the MAT A linked marker data sets, and (b) the ITS region. In both datasets DNA sequences were obtained from 83 isolates representing *S. lacrymans *and *S. himantioides*. Bayesian posterior probabilities above 0.50 are given above branches and Jackknife support values (1000 replicates) above 50 are given below branches. The tree symbols indicate isolates derived from a natural habitat (forest), otherwise the isolates were obtained from buildings. The star symbols indicate isolates with more than two alleles of the mating type linked marker while the arrows pinpoints trans-specific polymorphisms.

We found higher molecular variation in the mating type linked marker compared to the other sequenced loci (Table 1). Fig. 1 shows higher allelic variation in the mating type linked marker than in the ITS region. In the phylogenetic tree obtained from the mating type linked marker, the samples of var. *lacrymans *from Europe, North-America and Oceania (i.e. the 'Cosmopolitan group' [12], and the Japanese population appeared to a large extent intermixed on several branches. This is in contrast to the ITS tree (Fig. 1), as well as trees based on other analysed markers [12] where the Japanese and Cosmopolitan samples appear in two quite distinct groups. Furthermore, a comparable amount of genetic variation appeared in the mating type linked marker in populations of *S. lacrymans *from nature and buildings. This is in stark contrast to the pattern observed using neutral genetic markers, where natural populations were far more genetically variable than those from buildings (Table 1). The high level of genetic variation at the mating type linked marker compared to the presumably neutrally evolving markers may be explained by negative frequency-dependent selection at the MAT A locus maintaining genetic variation also in the vicinity of the locus under selection. In accordance with this hypothesis the results from the HKA test showed that there was significant deviation from neutral evolution when we compared the mating type linked marker with the presumably neutral markers ITS, *tub *or *gpd*. This test was based on intra- and interspecific divergence within and between *S*. *lacrymans *var. *shastensis *and var. *lacrymans*. Balancing selection, such as negative frequency-dependent selection, will often tend to keep alleles at intermediate frequencies. Accordingly, rare alleles will tend to be underrepresented compared to neutral expectation. Thus neutrality tests, such as Tajima's D will often yield positive test statistics at such loci. In contrast, the test statistics of Tajima's D, Fu and Li's D (Table 1) were negative at the mating type linked marker in the populations investigated here. In a previous study we found strong signals for a recent population expansion in these fungi [12], a demographic process that usually would affect the test-statistics of these neutrality tests in the opposite direction compared to balancing selection. Hence, we suggest that demographic effects may override the expected signal from balancing selection in this case.

Negative frequency-dependent selection counteracts the sorting effect of genetic drift, and may maintain a high level of molecular and allelic variation also in neighbouring linked regions. Previous studies has shown that tight genetic linkage to loci subjected to strong negative frequency-dependent selection will maintain high levels of polymorphism also at otherwise neutrally evolving loci [27,28]. Thus, we find it likely that the high level of genetic variation at our mating type linked marker is due to associated effects of negative frequency-dependent selection at MAT A, and not because it itself is subject to negative frequency-dependent selection itself.

### Species phylogeny

Although we find high levels of molecular variation at the mating type linked marker, the alleles largely group in accordance with known species delimitations [12]. All alleles obtained from the *S. himantioides *morphospecies cluster together (Fig. 1a) and all alleles from var. *lacrymans*, except a few trans-species polymorphisms (see below) occur in one group. However, alleles from the var. *shastensis *group separate into two different sister groups. Four trans-specific polymorphisms are observed, indicated by arrows in Fig. 1a. One sequence derived from a var. *shastensis *isolate (SHA8-1) clusters within the var. *lacrymans *group. On the opposite, one sequence obtained from a European var. *lacrymans *isolate (SL189) clusters within var. *shastensis *and another sequence obtained from the same isolate (SL189) clusters within *S. himantioides*. Lastly, one North American isolate of var. *lacrymans *(SL403) groups within *S. himantioides*. Notably, from SL189 and SL403, four and three different alleles were obtained (indicated by stars in Fig. 1a). The very same isolates are heterozygous at ITS and include two divergent haplotypes (Fig. 1b), of which one has a divergent placement according to geographic origin. One SL189 ITS allele clusters with Asian isolates and SL403 has a unique ITS haplotype. These peculiar observations can be explained in various ways. Ancient gene duplications events might be maintained in the genome of some or all isolates. If so, it should be expected that the trans-specific alleles cluster as more early diverging lineages in the respective groups. Alternatively, SL189 and SL403 could represent multiple heterokaryotic hybrids, including more than two karyotypes, which may account for the high number of alleles and divergent ITS haplotypes. It has been documented that the mycelia of other basidiomycetes can include more than two different nuclei [29]. However, it seems rather unlikely that the German isolate SL189 has acquired a copy from var. *shastensis *that mainly lives in California. More research is definitively needed to investigate possible scenarios for these puzzling observations.

### Geography

We would expect relatively little correspondence between geographic and genetic distances in a DNA region linked to a locus under strong selection [30]. However, some geographic structure can be observed in Fig. 1a. The European sequences clustered mainly into three main groups that might correspond to three MAT A alleles (see discussion below). Notably, within these three groups the sequences were either identical or very similar, only including some autapomorphic mutations. The distribution of European alleles corresponds well with other results indicating that the European population has recently expanded [13]. Several strictly Japanese sub-groups appeared as well. Notably, the Japanese alleles were in general more divergent than the European ones. This also corresponds well with the observation that higher genetic variation occurs in Japan in microsatellites, AFLPs and four sequenced loci [12,13]. Interestingly, alleles from the Asian mainland population, where var. *lacrymans *also has a natural distribution in forests, were distributed throughout the var. *lacrymans *part of the tree. In [12] it was observed that the natural Asian population included most of the genetic variation observed in the indoor founder populations, and this seems also to be the case for the MAT A-linked alleles.

### Dikaryons

In cases where two alleles of the mating type linked marker were amplified from the same dikaryon, these invariably turned out to be divergent, which is to be expected due to the linkage to MAT A. We found the two alleles of dikaryons to be significantly more divergent than expected by chance in the European population (p-value = 0.003), in the Cosmopolitan group (p-value = 0.010) and in all samples of var. *lacrymans *taken together (p-value = 0.017). However, we found no significant elevation of divergence in the Japanese population when analysed alone (p-value = 0.905), nor in var. *shastensis *(p-value = 0.678). These differences between the populations may be due to different number of mating type alleles present in the various groups. When a high number of mating types occur in a genetically variable population, which is likely to be the case for the Japanese population and var. *shastensis*, most primary mycelia will be able to form a dikaryon, resembling panmictic conditions. However, in the European population and the Cosmopolitan group as a whole, a limited number of mating types is likely to occur (see [31]), which means that only a portion of the primary mycelia will be able to mate. Selection is likely to favour individuals with different mating types in a genetically bottlenecked population, whereas random association of mating types is more likely in a genetically variable population. Alternatively, because linkage to mating type loci under strong balancing selection can create a scenario in which degeneration of the linked genes occurs [28], the preponderance of dissimilar *mip *alleles within dikaryons of var. *lacrymans *could be due to the phenomenon of associative overdominance due to recessive deleterious alleles at MAT linked loci [32,33]). This could be reinforced if the number of MAT alleles in the populations is small and the alleles are maintained in the populations for longer periods [33]. However, *S. lacrymans *has a free-living haploid stagen and genetic load due to exposure of recessive mutations would thus be reduced. Tentatively, therefore, we suggest that the former hypothesis of different strengths of selection for divergent alleles in populations with different levels of genetic variation seems more plausible than that of associative overdominance.

## Conclusions

In this study we use a mating type linked marker as a proxy to infer variation of mating types in MAT A in *S. lacrymans*. We present evidence consistent with the MAT A region being influenced by frequency-dependent selection, favouring rare alleles. Although analysis of a locus linked to a gene of interest may provide important information about the target gene, as argued here, one problem is that mutations at the analysed marker may muddle correct assignment of alleles at the gene of interest. For instance, we observed three main groups of sequences in the European population that may correspond to three MAT A alleles, but with some sequence variation within the groups, possibly caused by novel mutations at the analysed marker. To reveal the actual connection between the sequenced alleles and the mating types segregation analyses of spore families with mating types known from crossing experiments should be conducted.

## Authors' contributions

IBE carried out molecular genetic lab work, phylogenetic analyses and analyses of molecular evolution and drafted and wrote parts of the manuscript. IS carried out bioinformatics and statistical analyses and wrote parts of the manuscript. G-PS carried out analyses of molecular evolution and wrote parts of the manuscript. HK conceived of the study, and participated in its design and coordination, drafted and wrote parts of the manuscript. All authors read and approved the final manuscript.

## Supplementary Material

Additional file 1 **Analyzed material**. Information about GenBank accession numbers, geographic origin and ecology (from building or nature) of the analyzed specimens. Click here for file
