# Supplementary material for: High variability in a mating type linked region in the dry rot fungus Serpula lacrymans caused by frequency-dependent selection?
Source: BMC Genet. 2010 Jul 12;11:64. doi: 10.1186/1471-2156-11-64 (PMC2909151; doi:10.1186/1471-2156-11-64)
Supplement: Additional file 1 — Analyzed material. Information about GenBank accession numbers, geographic origin and ecology (from building or nature) of the analyzed specimens. [file 1471-2156-11-64-S1.PDF]

Additional file 1: Information about the analyzed material.

**Isolate**	**# of alleles**	**Genbank accession nos.**				**Origin**	**Ecology**
		**mip**	**ITS**	***tub***	***gpd***		
Cz1	1	HM022563	HM022435	HM022516	HM022473	Czech Republic; Jihlava	Nature
Cz2	1	HM022564	HM022436	HM022517	HM022474	Czech Republic; Jihlava	Nature
SL1	1	HM022565	GU196448	GU196680	GU196599	Norway	Building
SL2	1	HM022566	AJ518882	AM494524	AM494527	Norway; Oslo	Building
SL3	2	HM022567, HM022569	GU196449	GU196681	GU196600	Germany; Bad Bevensen	Building
SL4	1	HM022569	GU196450	GU196682	GU196601	Germany; Rothenburg	Building
SL5	1	HM022570	GU196451	GU196683	GU196602	Germany; Berlin	Building
SL83	1	HM022571	AJ518887	HM022518	HM022475	Belgium	Building
SL85	2	HM022572, HM022573	AJ518889	HM022519	HM022476	Belgium	Building
SL99	1	HM022574	AJ557309	HM022520		China; Nei Mongol	Building
SL108	1	HM022575	AJ557268			Canada; Ottawa; Ontario	Building
SL109	1	HM022576	AJ557273	HM022521	HM022477	Czech Republic; Plzen	Building
SL114	1	HM022577	AJ557275	HM022522		Czech Republic; Plzen	Building
SL116	1	HM022578	HM022437	HM022523		Czech Republic; Plasnice	Building
SL146	1	HM022579	AJ557276	HM022524		Czech Republic	Building
SL153	1	HM022580	AJ557269	HM022525		Canada; Ontario	Building
SL154	1	HM022581	AJ557370	HM022526		USA: New York	Building
SL161	1	HM022582	AJ518895	HM022527	HM022478	Norway; Haugesund	Building
SL164	1	HM022583	AJ518898	HM022528	HM022479	Norge: Rennebu	Building
SL182	1	HM022584	HM022438	HM022529	HM022480	England: Hampton Hill, Middx	Building
SL183	1	HM022585	HM022439	HM022530	HM022481	Canada: Fredericton	Building
SL184	2	HM022586, HM022587	HM022440	HM022531		Russia	Building
SL185	1	HM022588	HM022441	HM022532	HM022482	Russia	Nature
SL187	1	HM022589	HM022442	HM022533	HM022483	UK: Liverpool	Building
SL188	1	HM022590	HM022443	HM022534	HM022484	England: Liverpool	Building
SL189	4	HM022591, HM022592, HM022593, HM022594	HM022444	HM022535	HM022485	Germany: Eberswalde	Building
SL200	2	HM022595, HM022596	AJ419910	HM022536	HM022486	Poland; Warsaw	Building
SL201	2	HM022597, HM022598	HM022445	HM022537	HM022487	Australia: Ballarat	Building
SL202	1	HM022599	HM022446	HM022538	HM022488	France; Xylochimie	Building
SL204	2	HM022600, HM022601	HM022447	HM022539	HM022489	UK; Glasgow	Building
SL218	2	HM022602, HM022603	HM022448	HM022540	HM022490	Finland: Porvoo	Building
SL219	2	HM022604, HM022605	HM022449	HM022541	HM022491	Finland; Helsinki	Building
SL220	1	HM022606	HM022450	HM022542	HM022492	Finland; Manlyharju	Building
SL226	1	HM022607	HM022451	HM022543	HM022493	Russia	Nature
SL228	2	HM022608, HM022609	HM022452	HM022544	HM022494	Belgium; Baisy-Thy	Building
SL229	2	HM022610, HM022611	HM022453	HM022545	HM022495	New Zealand	Building
SL230	2	HM022612, HM022613	HM022454	HM022546	HM022496	New Zealand; Rotorua	Building
SL266	1	HM022614	HM022455	HM022547	HM022497	France; Normandie; Rouen	Building
SL267	1	HM022615	HM022456	HM022548	HM022498	Belgium, Brabant Wallon	Building
SL270	2	HM022616, HM022617	HM022457			Russia; Krasnoyarski krai	Nature
SL271	2	HM022618, HM022619	HM022458	HM022549		Russia; Kamchatka; Zhupanovo	Building

Isolate	# of alleles	Genbank accession nos. mip	ITS	*tub*	*gpd*	Origin	Ecology
SL272	1	HM022620	HM022459	HM022550		Russia; Krasnoyarski krai	Building
SL273	2	HM022621, HM022622	HM022460	HM022551		Russia; Kamchatka; Zhupanovo	Building
HIM28	2	HM022623, HM022624	AF335274	HM022552	HM022499	Himalaya	Nature
HIM67	2	HM022625, HM022626	AF335273	HM022553	HM022500	Himalaya	Nature
SL400	1	HM022627	HM022461	HM022554	HM022501	Canada	Building
SL403	3	HM022628, HM022629, HM022630	HM022462	HM022555	HM022502	Canada	Building
0201	2	HM022631, HM022632	GU196442	GU196674	GU196593	Japan	Building
0202	2	HM022633, HM022634	GU196443	GU196675	GU196594	Japan	Building
0203	1	HM022635	GU196444	GU196676	GU196595	Japan	Building
0204	1	HM022636	GU196445	GU196677	GU196596	Japan	Building
0301	2	HM022637, HM022638	GU196446	GU196678	GU196597	Japan	Building
0302	2	HM022639, HM022640	GU196447	GU196679	GU196598	Japan	Building
SL198	2	HM022641, HM022642	GU196372	GU196605	GU196524	Japan; Asahikawa	Building
SL199	2	HM022643, HM022644	GU196373	GU196606	GU196525	Japan; Asahikawa	Building
SL261	2	HM022645, HM022646	GU196374	GU196607	GU196526	Japan; Asahikawa	Building
SL262	2	HM022647, HM022648	GU196375	GU196608	GU196527	Japan; Kimobetsu	Building
SL263	1	HM022649	GU196376	GU196609	GU196528	Japan; Asahikawa	Building
SL264	2	HM022650, HM022651	GU196377	GU196610	GU196529	Japan; Sapporo	Building
SL265	2	HM022652, HM022653	GU196378	GU196611	GU196530	Japan	Building
SL268	1	HM022654	GU196379	GU196612	GU196531	Japan; Asahikawa	Building
SL269	2	HM022655, HM022656	GU196380	GU196613	GU196532	Japan; Nara	Building
SL393	1	HM022657	GU196383	GU196616	GU196535	Japan; Ina	Building
SHA8-1	2	HM022658, HM022659	AM494515	AM494517	AM494534	USA; California	Nature
SHA10-2	2	HM022660, HM022661	HM022463	AM494519	AM494538	USA; California	Nature
SHA10-3	2	HM022662, HM022663	HM022464	HM022556	HM022503	USA; California	Nature
SHA11	2	HM022664, HM022665	HM022465	AM494520	AM494535	USA; California	Nature
SHA17-1	2	HM022666, HM022667	HM022466	HM022557	HM022504	USA; California	Nature
SHA19	2	HM022668, HM022669	HM022467	AM494521	HM022505	USA; California	Nature
SHA20-1	2	HM022670, HM022671	HM022468	HM022558	HM022506	USA; California	Nature
SHA20-2	2	HM022672, HM022673	HM022469	AM494522	AM494536, AM494537	USA; California	Nature
SHA21-2	2	HM022674, HM022675	HM022470	HM022559	HM022507	USA; California	Nature
SHA29-1	1	HM022676	HM022471	AM494523	AM494533	USA; California	Nature
SHA30-2	1	HM022677	HM022472	HM022560	AM494539	USA; California	Nature
USA94	1	HM022678	AF335276	HM022561	HM022508	USA; California	Nature
HHB	1	HM022679	AM076493	HM022562	AM494540	USA; Alaska; Kenai Peninsula	Nature
SH20	1	HM022680	AM076492	AJ557382	HM022509	Unknown	Unknown
SH17	2	HM022681, HM022682	AM076494	AJ557380	HM022510	Canada; British Colombia	Nature
ThNor	2	HM022683, HM022684	AM076497	AM076414	HM022511	Norway	?
SH26	1	HM022685	AM076506	AJ557373	HM022512	Zimbabwe, Manicaland	Nature
SH99	1	HM022686	AM076514	AM076432	HM022513	USA; Madison	Unknown
SL86	1	HM022687	AJ557364	AM076415	HM022514		Building
SH18	1	HM022688	AM076499	AJ557377	HM022515	Canada, British Colombia	Nature
